# Deletion of *Kcnj16* in Mice Does Not Alter Auditory Function

**DOI:** 10.3389/fcell.2021.630361

**Published:** 2021-02-22

**Authors:** Jun Lv, Xiaolong Fu, Yige Li, Guodong Hong, Peipei Li, Jing Lin, Youfang Xun, Lucheng Fang, Weibin Weng, Rongyu Yue, Geng-Lin Li, Bing Guan, He Li, Yideng Huang, Renjie Chai

**Affiliations:** ^1^Department of Otolaryngology-Head and Neck Surgery, First Affiliated Hospital of Wenzhou Medical University, Wenzhou, China; ^2^State Key Laboratory of Bioelectronics, School of Life Sciences and Technology, Jiangsu Province High-Tech Key Laboratory for Bio-Medical Research, Southeast University, Nanjing, China; ^3^School of Life Sciences and Key Laboratory of the Ministry of Education for Experimental Teratology, Shandong University, Jinan, China; ^4^Waksman Institute, Rutgers, The State University of New Jersey, Piscataway, NJ, United States; ^5^Department of Otolaryngology, Head and Neck Surgery, Xiangya School of Medicine, Central South University, Changsha, China; ^6^Department of Otolaryngology, Head and Neck Surgery, Clinical Medical College of Yangzhou University, Yangzhou, China; ^7^Department of Otolaryngology-Head and Neck Surgery, Provincial Hospital Affiliated to Shandong University, Jinan, China; ^8^Department of Otorhinolaryngology and ENT Institute, Eye and ENT Hospital, Fudan University, Shanghai, China; ^9^Department of Otolaryngology-Head and Neck Surgery, Hwa Mei Hospital, University of Chinese Academy of Sciences, Ningbo, China; ^10^Co-Innovation Center of Neuroregeneration, Nantong University, Nantong, China; ^11^Institute for Stem Cell and Regeneration, Chinese Academy of Sciences, Beijing, China

**Keywords:** Kir5.1, hair cell, endolymphatic potential, cochlea, hearing loss

## Abstract

Endolymphatic potential (EP) is the main driving force behind the sensory transduction of hearing, and K^+^ is the main charge carrier. Kir5.1 is a K^+^ transporter that plays a significant role in maintaining EP homeostasis, but the expression pattern and role of Kir5.1 (which is encoded by the *Kcnj16* gene) in the mouse auditory system has remained unclear. In this study, we found that Kir5.1 was expressed in the mouse cochlea. We checked the inner ear morphology and measured auditory function in *Kcnj16*^–/–^ mice and found that loss of *Kcnj16* did not appear to affect the development of hair cells. There was no significant difference in auditory function between *Kcnj16*^–/–^ mice and wild-type littermates, although the expression of *Kcnma1*, *Kcnq4*, and *Kcne1* were significantly decreased in the *Kcnj16*^–/–^ mice. Additionally, no significant differences were found in the number or distribution of ribbon synapses between the *Kcnj16*^–/–^ and wild-type mice. In summary, our results suggest that the *Kcnj16* gene is not essential for auditory function in mice.

## Introduction

**FIGURE 1:**
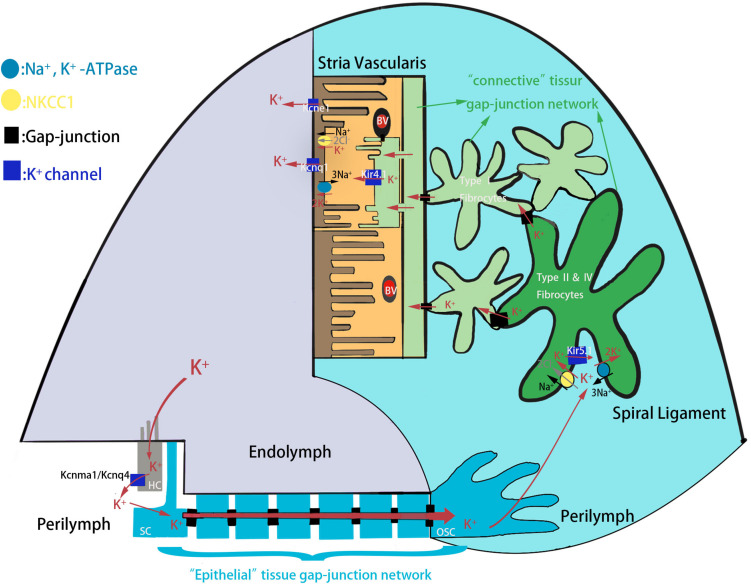
The schematic model of K^+^ circulation in mouse cochlea. K^+^ exits from HCs into the perilymph and is reabsorbed by the SCs and is further transported to the spiral ligament of the cochlear lateral wall through epithelial tissue gap-junction network. K^+^ is then reabsorbed by type II and IV fibrocytes, and recycled to the stria vascularis where it is finally secreted into the endolymph. HC, hair cell; SC, supporting cell; OSC, outer sulcus cell; BV, blood vessels.

Congenital hearing loss is one of the most prevalent sensory deficiencies in children and affects 1 in 500 newborns in developed countries, and hereditary factors account for the majority of cases (Morton and Nance, 2006; Korver et al., 2017). External sounds, which are transmitted through the external ear and middle ear, stimulate the cochlea, which is the sole hearing-related sensory organ in the inner ear. The cochlea contains two types of sensory hair cells (HCs)–including one row of inner hair cells (IHCs) and three rows of outer hair cells (OHCs)–and a variety of support cells (SCs) consisting of Deiters’ cells, pillar cells, Hensen’s cells, inner border cells, and inner phalangeal cells (Fettiplace, 2017; Li et al., 2018). The apical surfaces of HCs are exposed to endolymph, an extracellular fluid with 150 mM K^+^ and a potential of +80 mV that is produced by the stria vascularis, while the cell bodies are surrounded by the perilymph with 5 mM K^+^ and a potential of 0 mV (Wangemann, 2006; Kazmierczak et al., 2015; Nin et al., 2016). When the sound-driven vibration stimulates the basilar membrane, the hair bundles of HCs are deflected thus opening mechano-sensitive channels at the top of the stereocilia. This allows the influx of the endolymphatic K^+^ that excites the HCs to convert sound-driven vibrations into electrochemical signals that are transmitted to the brain along the auditory nerve for processing (Hudspeth, 1997; Kazmierczak and Müller, 2012). The K^+^ exits from HCs into the perilymph and is reabsorbed by the SCs and is further transported through the spiral ligament of the cochlear lateral wall, a connective tissue comprising several types of fibrocytes, to the stria vascularis where it is finally secreted into the endolymph (Figure 1). This circulation maintains a high K^+^ concentration and potential in the endolymph in order to provide the driving force for HC transduction. Interference with any step in the K^+^ circulation pathway of the cochlea will break the endolymphatic potential (EP) and result in hearing loss (Hibino and Kurachi, 2006; Nin et al., 2012).

Recent studies have shown that several ion-transport mechanisms play specialized roles in cochlear K^+^ circulation and in hearing. *Kcnq4* encodes an M-type K^+^ channel that is expressed in cochlear HCs, and mutations and targeted ablation of *Kcnq4* cause a form of non-syndromic dominant deafness referred to as DFNA2, which is characterized by the slow degeneration of OHCs and a disruption of the I_*K,n*_ current (Kharkovets et al., 2006), suggesting that K^+^ transporters are essential for hearing. Administration of Ba^2+^, a non-specific blocker of inwardly rectifying K^+^ channels, to the perilymph slightly elevated the EP (Kakigi et al., 2002), and thus, Ba^2+^-sensitive K^+^ channels are also believed to be involved in K^+^ homeostasis. As previously reported, Kir4.1 (which is encoded by the *Kcnj10* gene) is specifically distributed in the stria vascularis, cochlear ganglia, and Deiters’ cells and participates in generating and maintaining a positive EP in the endolymph. Missense and nonsense mutations of *Kcnj10* have been identified in EAST/SeSAME syndrome, which is characterized by epilepsy, ataxia, sensorineural deafness, renal tubulopathy, mental retardation, and electrolyte imbalance (Marcus et al., 2002; Sala-Rabanal et al., 2010; Chen and Zhao, 2014). *Kcnj16* mutations have been found in patients with non-familial Brugada syndrome, which manifests as cardiac arrhythmias and sudden death (Juang et al., 2014). In the mouse genome, Kir5.1 is encoded by a single exon of the *Kcnj16* gene, and the Kir5.1 protein often forms heteromeric inwardly rectifying potassium channels with Kir4.1 in many tissues, including Müller cells, renal epithelial cells, and locus coeruleus neurons (Lourdel et al., 2002; Ishii et al., 2003). Some studies have reported that deletion of *Kcnj16* in mice can impair renal function in excreting potassium during times of increased dietary potassium intake and can thus result in hypokalemia (Paulais et al., 2011; Wu et al., 2019). The locus coeruleus in *Kcnj16*^–/–^ mice has a dramatically reduced and delayed response to cytoplasmic alkalinization and acidification, suggesting that Kir5.1 is an important determinant of *P*CO_2_/pH sensitivity in locus coeruleus neurons (D’Adamo et al., 2011; Puissant et al., 2017). In the cochlea, Kir5.1 is expressed in type II, IV, and V fibrocytes in the lateral wall (Hibino et al., 2004), and *Kcnj16* mRNA as well as Kir5.1 protein decrease with age (Pan et al., 2016), suggesting that Kir5.1 might play an important role in the pathogenesis of age-related hearing loss.

In order to understand the role of Kir5.1 in the mouse auditory system, we generated *Kcnj16*^–/–^ mice using CRISPR-Cas9 technology and studied the expression of Kir5.1 in the cochlea and the role of the protein in auditory function using the *Kcnj16*^–/–^ mouse model.

## Results

### Localization of Kir5.1 in the Mouse Cochlea

**FIGURE 2:**
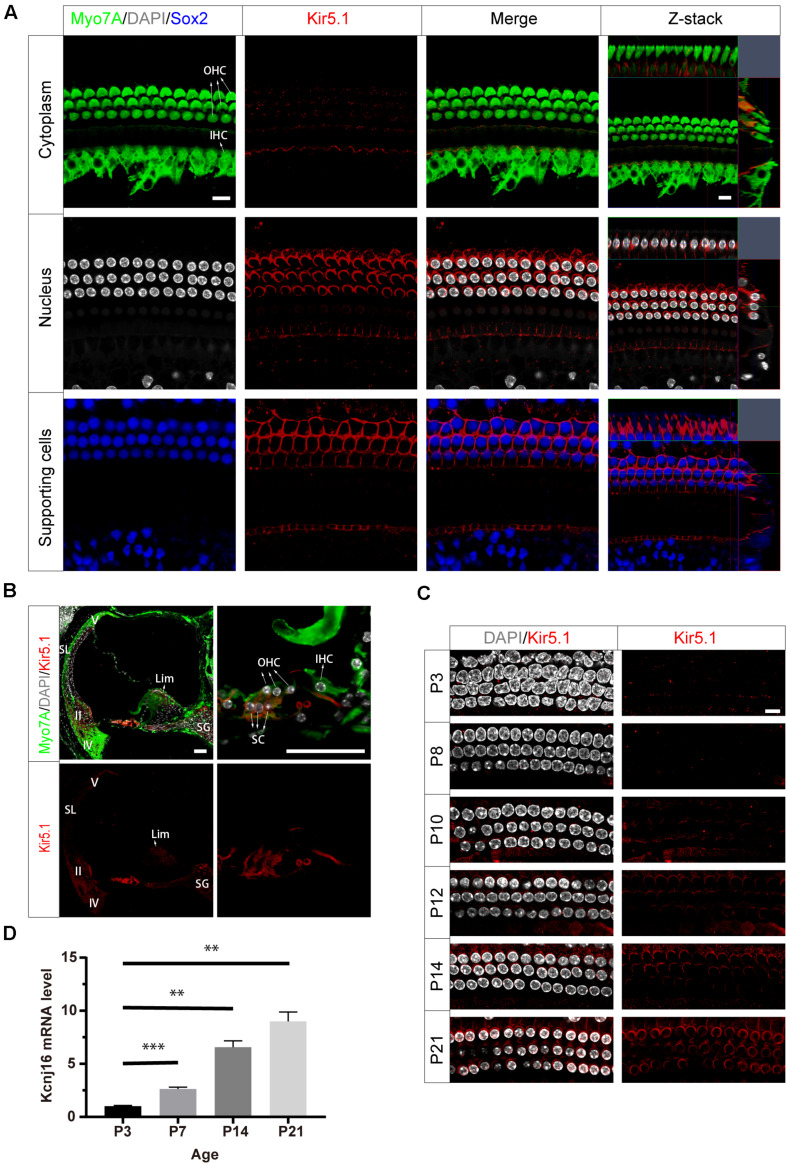
Expression of Kir5.1 in the WT mouse cochlea. **(A)** Immunofluorescence staining with antibodies against Kir5.1 (red), Myo7A (green), and Sox2 (blue) and DAPI staining (white) in the basal turn of the mouse cochlea at P90. There was no difference in the immunolabeling of Kir5.1 from the apical to basal turns. Scale bar = 10 μm. **(B)** Immunohistochemical staining with antibodies against Kir5.1 (red) and Myo7A (green) and DAPI staining (white). Scale bar = 50 μm. **(C)** Immunofluorescence staining with antibodies against Kir5.1 (red) and DAPI staining (white) in the cochlea at P3, P8, P10, P12, P14, and P21. Scale bar = 10 μm. **(D)** Q-PCR results showing the changes in *Kcnj16* mRNA in the mouse cochlea from P3 to P21. GAPDH was used as the internal control. Primers are shown in the Supplementary Table 2. Data are presented as the mean ± SD. ^∗∗^*p* < 0.01, ^∗∗∗^*p* < 0.001, *n* = 4. OHC, outer hair cell; IHC, inner hair cell; SC, supporting cell; SL, spiral ligament; II, IV, V, type II, IV, and V fibrocytes in the spiral ligament. Lim, spiral limbus; SG, spiral ganglions.

To investigate the localization of Kir5.1 in the mouse cochlea, we first immunolabeled Kir5.1 in the cochlear epithelia of postnatal day 90 (P90) wild-type (WT) mice with antibodies against Myo7A, a marker specifically expressed in HC cytoplasm, and antibodies against Sox2, which label the nuclei of SCs. DAPI was used to label the DNA. Confocal imaging of the whole-mount organ of Corti samples showed that Kir5.1 was mainly expressed in the cytomembrane of SCs and at the bottom of HCs (Figure 2A), while no difference was found in the immunolabeling of Kir5.1 from the apical to basal turns of the cochlea (data not shown). To further determine the expression of Kir5.1 in the cochlea, immunohistochemistry was performed using antibodies against Kir5.1 and Myo7A. We observed Kir5.1 immunoreactivity in the lower part of spiral ligament where type II and IV fibrocytes are found and in the suprastrial part of the spiral ligament where type V fibrocytes dominate. We also detected Kir5.1 immunoreactivity in the spiral limbus and spiral ganglions, and strong Kir5.1 immunoreactivity was detected in the membrane of outer SCs and the bottom of HCs. No immunoreactivity was found in outer sulcus cells or in the middle part of the ligament (Figure 2B). When looking at earlier time points, we found that the expression of Kir5.1 began at P10 and increased during development (Figure 2C and Supplementary Figure 1A). In order to better define the temporal expression of the *Kcnj16* gene, q-PCR was performed in the WT cochlea at P3, P7, P14, and P21. We observed that *Kcnj16* mRNA expression was significantly increased from P3 to P21 (Figure 2D).

### Generation of *Kcnj16*^–/–^ Mice

**FIGURE 3:**
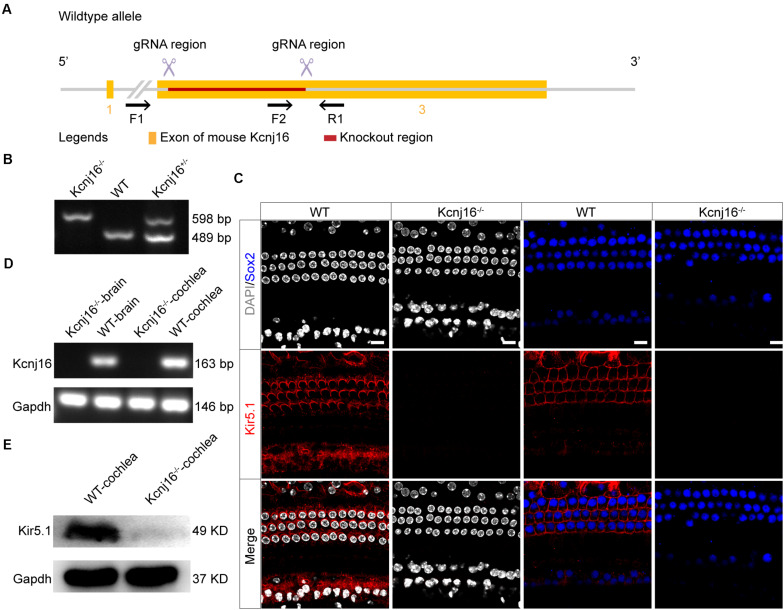
Analysis of Kir5.1 expression in *Kcnj16*^− /−^ mice at the protein and mRNA level. **(A)** Overview of the targeting strategy. **(B)** PCR results showing genomic DNA of a homozygous (*Kcnj16*^− /−^; 598 bp), a wild-type (WT; 489 bp), and a heterozygous (*Kcnj16*^+/−^; 489 and 598 bp) mouse. Primers are shown in the Supplementary Table 1. **(C)** The cochleae of P30 *Kcnj16*^− /−^ mice were immunolabeled with Kir5.1 antibodies. Scale bar = 10 μm. Images were taken from the middle turns of the sensory epithelium. There was no difference in the immunolabeling of Kir5.1 from the apical to basal turns (data not shown). **(D)** RT-PCR was performed to identify the presence the *Kcnj16* mRNA. RNA was extracted from the cochlea and from total brain tissue of P30 *Kcnj16*^− /−^ and WT mice, and GAPDH was used as the internal control. Primers are shown in the Supplementary Table 2. **(E)** Western blot was performed using antibodies against Kir5.1. Proteins from the cochlea were extracted from P30 *Kcnj16*^− /−^ and WT mice, and GAPDH was used as the internal control.

To investigate the specific role of Kir5.1 in the cochlea, we used CRISPR/Cas9 technology to generate *Kcnj16*^–/–^ mice. *Kcnj16* gene is located on Mouse chromsome 11 and 3 exons are identified. Two specific gRNAs were designed to target the exon 3 (Figure 3A). The pups were genotyped as *Kcnj16*^–/–^, *Kcnj16*^+/–^, and *Kcnj16*^+/+^ (WT) by PCR genotyping (Figure 3B) using the primers shown in Supplementary Table 1. To confirm whether the coding exon 3 of the *Kcnj16* gene was knocked out in the mice, we dissected the cochleae and brains from WT and *Kcnj16*^–/–^ mice at P30. The immunolabeling of Kir5.1 was seen in the membrane of SCs and at the bottom of HCs in the WT mouse cochleae, but almost no signal was detected in the *Kcnj16*^–/–^ cochleae (Figure 3C). The RT-PCR showed that the *Kcnj16* gene was knocked out in *Kcnj16*^–/–^ mice (Figure 3D). Western blot showed that a specific band, consistent with the expected size of Kir5.1 at 49 kDa, was found in WT mice, while no band was seen in *Kcnj16*^–/–^ mice (Figure 3E). These results confirmed that the *Kcnj16* gene was successfully knocked out in the *Kcnj16*^–/–^ mouse cochlea.

### No Changes Were Seen in General Cochlear Development or Stereocilia Structure in the *Kcnj16*^–/–^ Mice

**FIGURE 4:**
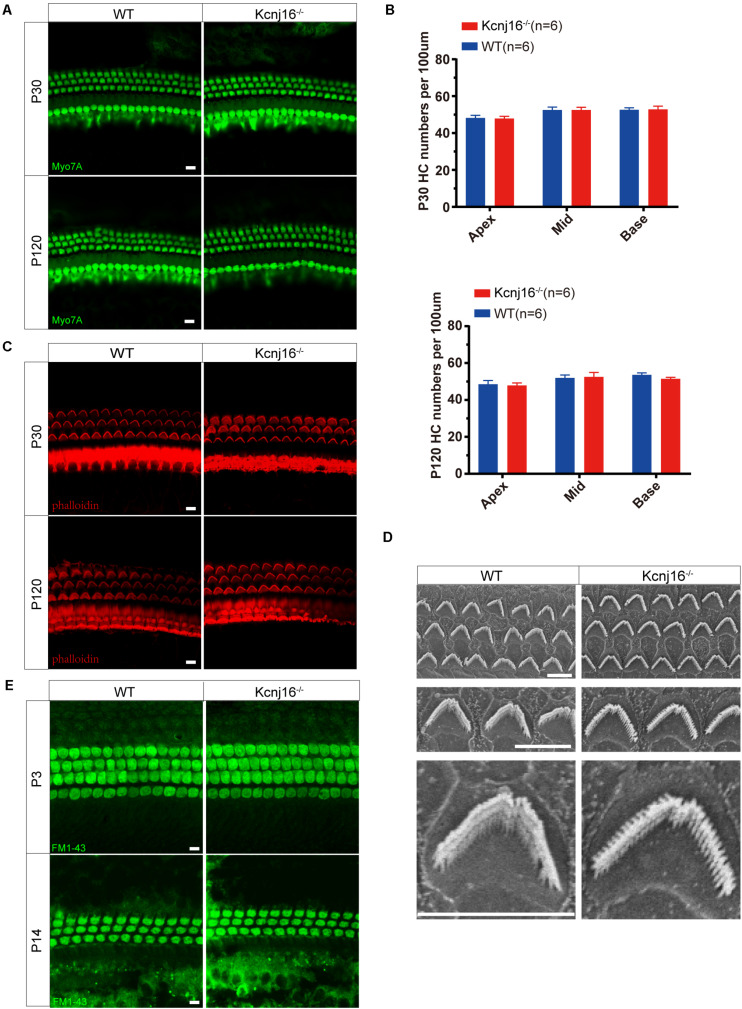
Cochlear development and stereocilia structures were normal in the *Kcnj16*^− /−^ mice. **(A)** Auditory HCs of P30 and P120 mice were stained with antibodies against Myo7A and imaged using a confocal microscope. Images were taken from the basal turn of the cochlea. There was no difference in the staining from the apical to basal turns. Scale bar = 10 μm. **(B)** The HCs were counted and compared with age-matched WT mice (*p* > 0.05, *n* = 6). Data are presented as the mean ± SD. **(C)** Auditory HC stereocilia of *Kcnj16*^− /−^ and WT mice were stained with phalloidin, and images were taken from the basal turn of the cochlea. There was no difference from the apical to basal turns. Scale bar = 10 μm. **(D)** Low magnification and high magnification scanning electron microscope images of OHC stereocilia bundles in *Kcnj16*^− /−^ and WT mice. Images were taken from the middle turn of P60 mice. Scale bar = 5 μm. **(E)** Auditory HCs of P3 and P14 mice were stained with FM1-43, and images were taken from the basal turns. There was no difference in the immunolabeling signals from the apical to basal turns (data not shown). Scale bar = 10 μm.

We next determined whether loss of Kir5.1 would influence the development of the sensory structures of the inner ear. To evaluate the survival of HCs, we stained whole-mount cochleae from *Kcnj16*^–/–^ and WT mice with the Myo7A antibody. Both IHCs and OHCs appeared normal in P30 *Kcnj16*^–/–^ mice compared with P30 WT littermates, and we did not observe any distinguishable HC loss in P30 *Kcnj16*^–/–^ mice. We did not detect any significant differences in morphology or HC populations between WT and *Kcnj16*^–/–^ mice, even at P120 (Figures 4A,B and Supplementary Figure 2A).

Sound detection by cochlear HCs depends on the deflection of the hair bundles, which are made up of actin-based stereocilia, in order to initiate the transformation of mechanical energy into electrical signals (Corns et al., 2018; Caprara et al., 2019; Pacentine et al., 2020). Therefore, we performed immunofluorescence staining of phalloidin and found that the stereocilia polarity of P60 cochlear HCs in *Kcnj16*^–/–^ mice was normal (Figure 4C and Supplementary Figure 2B). We then performed scanning electron microscopy to further examine the structure of the cochlear HC stereocilia from *Kcnj16*^–/–^ mice and age-matched WT mice. The cochlear sensory epithelium was normally patterned into one row of IHCs and three rows of OHCs, and stereocilia hair bundles exhibited the characteristic staircase-like arrangement (Figure 4D). These results suggest that deletion of *Kcnj16* does not affect the morphology of the cochlea or HC stereocilia in mice. FM1-43, a permeant blocker of the mechanotransduction channel (Gale et al., 2001), was used to investigate HC function in *Kcnj16*^–/–^ mice, and there was no significant difference between WT and *Kcnj16*^–/–^ mice at P3 or P14 (Figure 4E).

### *Kcnj16*^–/–^ Mice Showed No Changes in Auditory Function

**FIGURE 5:**
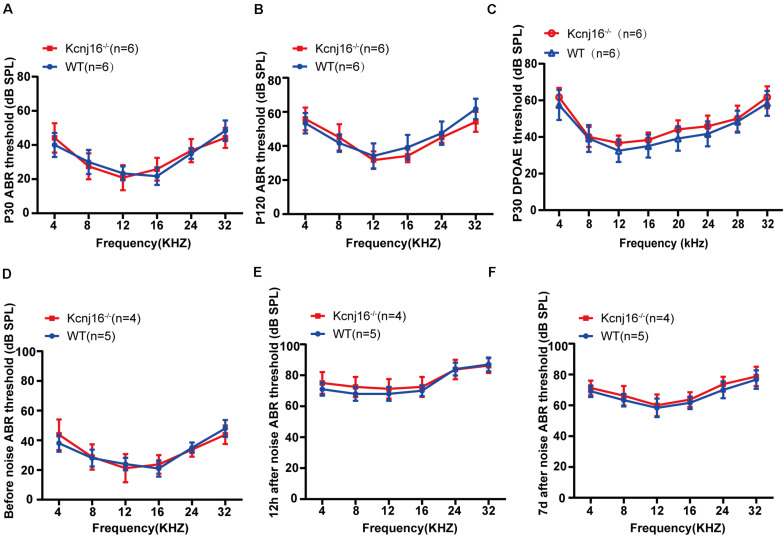
*Kcnj16*^− /−^ mice showed no changes in auditory function. **(A,B)** ABR thresholds of P30 and P120 *Kcnj16*^– /−^ and WT mice were measured at 4, 8, 12, 16, 24, 28, and 32 kHz. **(C)** DPOAE thresholds were measured in response to tone bursts of 4, 8, 16, 20, 24, 28, 32, 36, and 40 kHz in P30 *Kcnj16*^− /−^ and WT mice. **(D–F)** The ABR thresholds of P30 *Kcnj16*^− /−^ and WT mice were measured before, 12 h after, and 7 days after noise exposure. No significant differences were observed between *Kcnj16*^− /−^ and WT mice (*p* > 0.05). Data are presented as means ± SD.

Although, *Kcnj16*^–/–^ mice have been reported to be generally healthy (Lachheb et al., 2008), their auditory function has remained unknown. To investigate the contribution of *Kcnj16* defects to auditory function, we measured the auditory brainstem response (ABR) thresholds and found no difference between *Kcnj16*^–/–^ mice and WT controls at P30 or P120 (Figures 5A,B). Because ABR reflects the synchronized electrical activity of the ascending auditory pathway, we next measured the distortion-product otoacoustic emissions (DPOAEs), which reflect OHC activity. There was no difference in DPOAE threshold between P30 *Kcnj16*^–/–^ mice and WT littermates (Figure 5C). In order to investigate whether a stimulus would lead to observable differences between P30 WT and *Kcnj16*^–/–^ mice, we subjected the mice to noise exposure. The ABRs were measured before, 12 h after, and 7 days after noise exposure, and no significant difference was found between P30 WT and *Kcnj16*^–/–^ mice (Figures 5D–F). Together these results showed that *Kcnj16*^–/–^ mice have normal auditory function.

### The Expression of *Kcnma1*, *Kcnq4*, and *Kcne1* Is Decreased in P30 *Kcnj16*^–/–^ Mice

**FIGURE 6:**
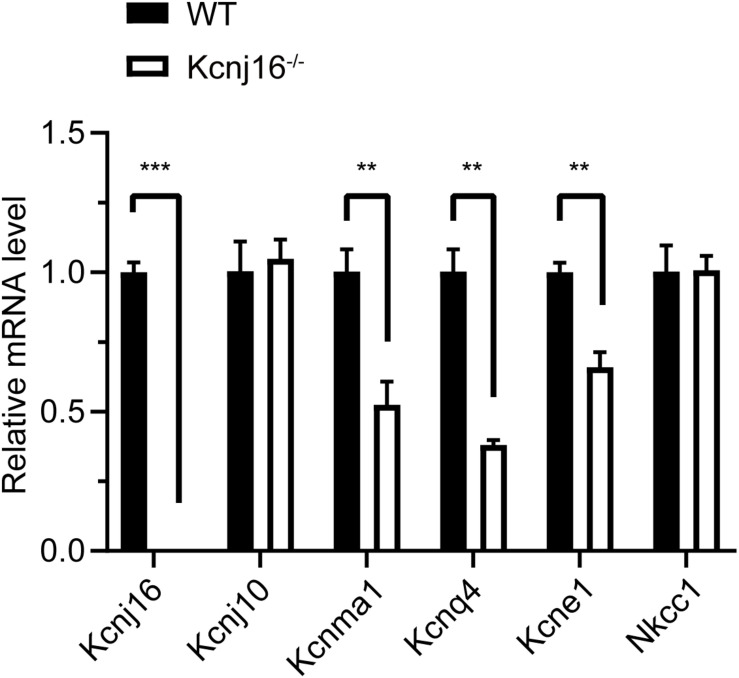
The expression of *Kcnma1*, *Kcnq4*, and *Kcne1* decreased in P30 *Kcnj16*^− /−^ mice. Q-PCR was performed with P30 WT and *Kcnj16*^− /−^ cochleae (*p* < 0.05, *n* = 4). Primers are shown in the Supplementary Table 2. Data are presented as the mean ± SD. **p < 0.01, ***p < 0.001, *n* = 4.

Because Kir5.1 subunits form a heteromeric assembly with Kir4.1 in the basolateral membrane of the mouse kidney (Lachheb et al., 2008) and because Kir4.1 (*Kcnj10*) is significantly upregulated in the renal cortex tissue of salt-sensitive *Kcnj16*^–/–^ mice (Palygin et al., 2017), we wondered if there were also any compensatory changes in Kir4.1 expression in the cochleae of *Kcnj16*^–/–^ mice. The q-PCR results showed there was no difference in *Kcnj10* expression between WT and *Kcnj16*^–/–^ mice, but we found that the expression of *Kcnma1*, *Kcnq4*, and *Kcne1*, which code for other K^+^ channel proteins in the cochlea, were significantly decreased in *Kcnj16*^–/–^ mice compared with WT mice, while *Nkcc1* showed no significant difference (Figure 6).

### The Morphology of IHC Ribbon Synapses Is Preserved in *Kcnj16*^–/–^ Mice

**FIGURE 7:**
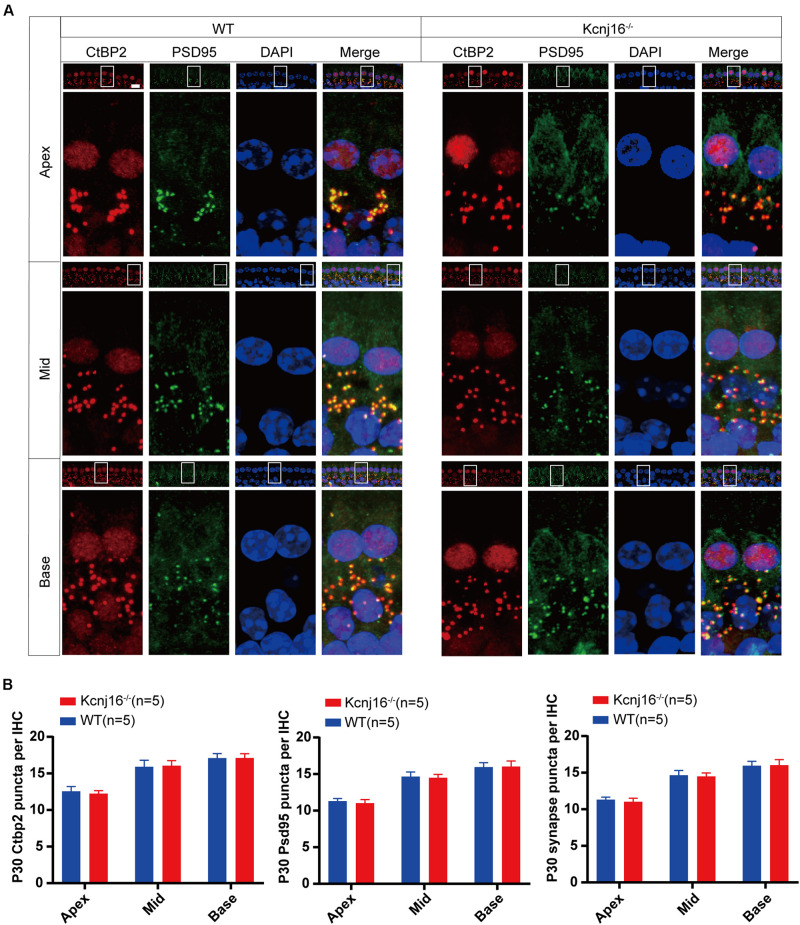
The morphology of IHC ribbon synapses is preserved in *Kcnj16*^− /−^ mice. **(A)** Ribbon synapses of P30 *Kcnj16*^− /−^ and WT mice were stained with the ribbon synapse-specific markers CtBP2 and PSD95 and imaged under a confocal microscope. Scale bar = 10 μm. **(B)** The total numbers of CtBP2 and PSD95 puncta and synapses from the apical to basal turns were counted and compared between WT and *Kcnj16*^− /−^ mice. No significant differences were seen for any measurements (*p* > 0.05, *n* = 5). Data are presented as the mean ± SD.

Ribbon synapses establish connections between IHCs and the afferent nerves of spiral ganglion neurons, and changes in ribbon synapses occur prior to IHC apoptosis. In noise-induced hearing loss, there is an acute loss of afferent nerve terminals, while cochlear HCs remain intact (Kujawa and Liberman, 2009; Li et al., 2016). Therefore, we evaluated the molecular composition and integrity of IHC ribbon synapses in the cochlea by whole mount staining with antibodies against CtBP2 and PSD95. As specialized components of the pre-synaptic ribbons, the ribbon synapses contain three transcriptional repressors: CtBP1, CtBP2, and an alternative splice form called RIBEYE. The CtBP2 signals were seen in the lateral aspects of the IHCs, and consistent with a previous study the PSD95 signal was concentrated at the terminus and the plasma membrane of IHCs (Marcus et al., 1985), and this staining did not interfere with the detection of the target signal. The red dot-like staining of CtBP2 at the base of the IHCs overlapped with the PSD95 signal, and there were comparable amounts of CtBP2 and PSD95-positive puncta in cochlear IHCs between *Kcnj16*^–/–^ and WT control mice, and no significant differences were seen in the synapse density of IHCs in *Kcnj16*^–/–^ mice compared to WT mice (Figures 7A,B). Because nerve fibers can’t trigger an action potential when pre-synapses are disconnected from post-synapses, we analyzed the co-localization of CtBP2 and PSD95. We found that almost all of the CtBP2 puncta were overlapping with PSD95 in both WT and *Kcnj16*^–/–^ mice. Thus, our results indicate that the morphology of IHC synapses were largely unaffected in *Kcnj16*^–/–^ mice.

## Discussion

In order to explore the role of *Kcnj16* in the mouse auditory system and in cochlear development, we generated *Kcnj16*^–/–^ mice. The ablation of *Kcnj16* did not affect auditory function, and no changes were seen in the overall development of the cochlea or ribbon synapses between the *Kcnj16*^–/–^ and WT mice, while the expression of *Kcnma1*, *Kcnq4*, and *Kcne1* was significantly decreased in the *Kcnj16*^–/–^ mice.

Kir5.1 subunits form a heteromeric assembly with Kir4.1 in the basolateral membrane of principal cells in the renal collecting duct (Lachheb et al., 2008) and in locus coeruleus neurons (D’Adamo et al., 2011). However, Kir5.1 and Kir4.1 are expressed in different regions in the mouse cochlear lateral wall, and Kir5.1 expressed in the cochlea might preferentially form complexes with other Kir5.1 proteins (Paulais et al., 2011). Consistent with a previous study (Hibino et al., 2004), our immunochemical experiments indicated that the Kir5.1 subunit is expressed in the lower part and suprastrial zone of the spiral ligament and in the spiral limbus and ganglions. However, we also found that Kir5.1 is expressed in the cytomembrane of SCs and at the bottom of HCs by whole-mount immunostaining. The difference in expression pattern might be because they used rats while we used mice, and it is also possible that the Kir5.1 protein is not highly conserved. Regardless of these differences, however, no immunoreactivity of Kir5.1 was detected in the *Kcnj16*^–/–^ mouse cochleae indicating that our antibody was effective.

The subcellular locations and structural arrangements of hair bundles and ribbon synapses in *Kcnj16*^–/–^ mice showed no changes compared to age-matched WT mice up to P120. However, it is possible that older *Kcnj16*^–/–^ mice might have more severe hearing loss compared to WT controls, and this needs to be further studied. Moreover, the effect of *Kcnj16* in the development of dendritic spines on auditory nerve dendrites was not investigated in the current study and should also be investigated in the future.

The endolymph of the cochlea contains 150 mM K^+^ and has a highly positive potential of +80 mV compared with perilymph, and this unique ionic and voltage environment is necessary for proper auditory function (Von Bekesy, 1952). K^+^ circulation is thought to maintain the EP through the cochlear lateral wall. Kir4.1 is the only Kir channel subunit expressed in the stria vascularis (Hibino et al., 1997), and administration of Ba^2+^ to the stria vascularis inhibits the maintenance of the EP (Marcus et al., 1985). Consistent with this, *Kcnj10*^–/–^ mice are profoundly deaf due to the absence of the EP and the loss of endolymphatic K^+^ (Marcus et al., 2002; Rozengurt et al., 2003). In *Kcnj10*^–/–^ mice, the spiral ganglion neurons and HCs show rapid degeneration after birth (Rozengurt et al., 2003), and most *Kcnj10*^–/–^ mice die by 3 weeks of age (Kofuji et al., 2000). Unlike *Kcnj10*^–/–^ mice and *Kcnj16*^–/–^ mice appear generally healthy and have proper auditory function and cochlear development. Several possible reasons might explain the differences between the two genotypes. First, when Kir4.1 is expressed alone in mice tissues it forms a tetramer and elicits a K^+^ current (Pessia et al., 1996; Kaiser et al., 2006), while Kir5.1 is non-functional when it is expressed alone in a heterologous expression system (Hibino et al., 2010). However, when Kir5.1 co-localizes with Kir4.1, they from a functional heteromer whose biophysical properties and sensitivity to intracellular H^+^ are different from those of the Kir4.1 homomer (Tanemoto et al., 2000; Tucker et al., 2000). Because Kir5.1 and Kir4.1 are distributed in distinct regions of the cochlea (Hibino et al., 2004), the Kir5.1 homomer in the cochlea might not be essential. Second, single-channel analysis of the *Kcnj10* mutation model revealed a strongly reduced mean open time, which was qualitatively similar to co-expression of Kir4.1/Kir5.1, suggesting the dominance of Kir4.1 function in native renal Kir4.1/Kir5.1 heteromers (Reichold et al., 2010; Williams et al., 2010). In addition, administration of Ba^2+^ and Cs^2+^, which are blockers that more selectively and preferentially inhibit Kir channels than Kv channels, to the perilymph only slightly increases the EP (Marcus, 1984; Wang et al., 1993; Takumi et al., 1995; Hibino et al., 2004). These results also suggest that Kir5.1 homomers might not be essential in the cochlea. Third, K^+^ channels, which are encoded by Kcnq4 and Kcnma1, function in hair cells by releasing K^+^ into the perilymph when HCs are excited. The K^+^/Cl^–^ co-transporters Kcc3 and Kcc4 that are expressed in Deiters’ cells absorb excess extracellular K^+^ and modulate the K^+^ circulation in the cochlea, and the Na^+^-K^+^-ATPase and the Na^+^-K^+^-2Cl^–^ co-transporter Nkcc1 in the spiral ligament contribute to the generation of the EP by facilitating cochlear K^+^ circulation in the ligament. Kir4.1, which is abundantly expressed in the stria vascularis, is another Kir channel protein that modulates the EP. Thus, these alternative K^+^ channel proteins might modulate the K^+^ circulation in the inner ear to maintain the EP and compensate for the lack of Kir5.1 proteins. Taken together, our data support the idea that Kcnma1, Kcnq4, and Kcne1 compensate for the lack of Kir5.1 (Figure 1).

In summary, our results demonstrate the expression patterns of Kir5.1 in the mouse cochlea. Because of the compensatory roles of Kcnq4, Na^+^-K^+^-ATPase, the Na^+^-K^+^-2Cl^–^ co-transporter Nkcc1, Kir4.1, and other potassium channel proteins, the loss of *Kcnj16* did not significantly change the number and function of ribbon synapses or the overall cochlear structure or auditory function.

## Materials and Methods

### Animals and Genotyping

All procedures were performed in accordance with research guidelines of the Institutional Animal Care and Use Committee (IACUC) at Southeast University and were in agreement with the National Institutes of Health Guide for the Care and Use of Laboratory Animals. Mice of both sexes were studied. For all experiments, control littermates had at least one wild-type allele of *Kcnj16*. All efforts were made to minimize the number of animals used and to reduce their suffering.

*Kcnj16*^–/–^ mice were generated using the CRISPR-Cas9 technology. Briefly, the *Kcnj16* gene is located on mouse chromosome 11 in which exon 3 was selected as the target site with the ATG start codon and the TAG stop codon. Two gRNAs (gRNA1, CGATGTCAGTTAAAAGTTCCAGG; gRNA2, TGGAGATCCTATTTAAAGTCAGG, designed by https://www.cyagen.com/cn/zh-cn/sperm-bank/16517) were designed to target the exon 3, which starts from about 0.08% of the coding region and covers 100% of the coding region. C57BL/6 background mice were chosen as the embryo donors. Cas9 and gRNA were co-injected into zygotes for *Kcnj16*^+/–^ mouse production. The zygotes were then transferred to the pseudopregnant CD1 female mice. The strain was backcrossed with C57BL/6J for more than five generations to establish an isogenic strain. Inter-cross the heterozygous mice to generate homozygous targeted mice. The pups were being genotyped by PCR of tail DNA, and the primers used for genotyping are shown in Supplementary Table 1. Potential off-target sites were predicted by an online database, and every potential off-target site was amplified by PCR followed by sequencing. No off-target sites were found.

### Immunohistochemistry

Whole-mount immunohistochemistry was performed as previously described (Fang et al., 2019). The cochleae were dissected from the temporal bones in cold PBS and were then fixed in 4% polyoxymethylene (Sigma,158127) for 1 h, followed by permeabilization with 0.1% Triton X-100 (Solarbio, T8200-500) for 1 h. After blocking (10% donkey serum, 0.5% Triton X-100, and 1% BSA in PBS at pH 7.2) for 1 h at room temperature, the sensory epithelia were incubated with the following primary antibodies overnight at 4°C: anti-Kir5.1 (Abnova, PBA-18407); anti-Myo7A (Proteus Bioscience, 25-6790; DSHB,138-1); anti-Sox2 (Santa Cruz Biotechnology, sc-17320); anti-CtBP2 (BD Biosciences, 612044); and anti-PSD95 (Millipore, MAB1596). The samples were washed three times with 0.1% Triton X-100 and incubated for 1 h at 37°C with the appropriate secondary antibody (Invitrogen: A31572, A21206, A31573, A31570, A21202, A31571, A21432, A11055, A21447, A21131, and A21240). Phalloidin (Thermo Fisher, A12379) was used to stain the actin cytoskeleton, and DAPI (Solarbio, C0060) was used to label the nuclei. Finally, the samples were washed three times again and mounted on glass slides with DAKO (DAKO, S3023). Whole-mount preparations were imaged using a confocal microscope (Zeiss LSM710).

AS for immunostaining of FM1-43. The cochleae were immersed in medium containing 3 mM FM1-43 (Thermo Fisher, F35355) for 45 s and washed three times in PBS. After 1 h in% polyoxymethylene, the samples were washed three times again and mounted on glass slides with DAKO followed by image in a confocal microscope.

Slice immunohistochemistry was performed as reported previously (Higashi et al., 2001; Ishii et al., 2003). Briefly, after sample preparation, the cryosections of the cochlea (10 μm) were incubated with anti-Kir5.1 and anti-Myo7A antibodies. The sections were then incubated with DAPI and the appropriate secondary antibody after washing. The samples were examined under a confocal microscope.

### Auditory Measurement

At 1–4 months of age, mice were anesthetized using 10 mg/kg pentobarbital sodium by intraperitoneal injection and kept on a thermostatic heating pad at 38°C. Three subdermal electrodes were placed at the vertex (recording), ipsilateral mastoid (reference), and contralateral hind limb (ground). A TDT System III workstation running SigGen32 software (Tucker-Davis Technologies, United States) was used to generate tone burst stimuli at 4, 8, 12, 16, and 32 kHz (Han et al., 2016; Pan et al., 2017). For each frequency, the sound level was decreased in 5-dB steps from 90 to 10 dB SPL. The hearing threshold was defined as the lowest level to produce a reproducible ABR response. The stimulus (10 ms tone bursts for ABR with a 0.5 ms rise/fall time and cos2 gating) were emitted by a broadband speaker (MF1; TDT) that was placed 10 cm in front of the mouse’s head.

The DPOAE was measured as previously described (Schwander et al., 2007; Kazmierczak et al., 2017). Briefly, the BioSig software and TDT hardware were used to generate two primary tones (f1 and f2) with a ratio of f2/f1 = 1.25. Equal stimulus levels (60 dB SPL) were coupled into the ear canal via a custom-made probe containing an MKE-2 microphone. DPOAEs were recorded at 4, 8, 12, 16, 20, 24, 28, 32, 36, and 40 kHz, and the amplitudes of the cubic distortion tone (2f1–f2) were analyzed by fast Fourier transformation.

Noise exposure was performed in an Industrial Acoustics double-walled soundproof room. Each fully awake mouse at P30 was positioned in a 9 × 9 × 9 cm wire cage and exposed to 8–16 kHz octave band noise at 118 dB SPL for 4 h (Ohlemiller et al., 2000). Noise was generated by custom Labview routines and presented using a TDT RZ6 combined with a Crown Audio power amplifier. ABR thresholds were measured before, 12 h after, and 7 days after noise exposure.

### Scanning Electron Microscopy

Scanning electron microscopy was performed as previously reported (Kazmierczak et al., 2017). The cochleae from P60 mice were fixed in 2.5% glutaraldehyde (Sigma-Aldrich, G5882) in 0.1 M phosphate buffer (pH 7.4) overnight at 4°C and then decalcified for 4 h in 0.5 M EDTA (Biosharp, BL518A). Samples were stored and shipped in 0.1% glutaraldehyde and then dehydrated through ethanol and critically point dried with liquid CO_2_ (CPD300, Leica). After coating with gold, the cochlear samples were imaged on a scanning electron microscope (Quanta 250 FEG).

### RT-qPCR

Total cochlear RNA was extracted with Trizol Reagent (Life, 15596-018), and cDNA was obtained using the Revert Aid First Strand cDNA synthesis kit according to the manufacturer’s instructions (Thermo Fisher Scientific, K1622). Quantitative PCR was performed on an Applied Biosystems CFX96 real-time PCR system (Bio-Rad, Hercules, CA, United States) with the SYBR Green (Rox) q-PCR Master Mix (Roche Life Science, 04913850001). The q-PCR conditions were set as follows: 15 s denaturation at 95°C followed by 40 cycles of 15 s denaturation at 95°C, 60 s annealing at 60°C, and 20 s extension at 72°C. The value for GAPDH was used to normalize the mRNA expression, and the results were calculated using the comparative cycle threshold (ΔΔCt) method. Primers used for q-PCR are shown in Supplementary Table 2.

### Western Blot

Cochleae from around 10 mice were dissected in cold PBS and lysed with RIPA lysis buffer (Protein Biotechnology, PP109) and protease inhibitor cocktails (Roche, 4906845001) at 4°C. The protein concentration was measured by a BCA protein quantification kit (Protein Biotechnology, PP20). Equal amounts of protein were fractionated by 10% SDS-PAGE gel and transferred to a polyvinylidene fluoride membrane. After blocking in 5% milk, the membrane was incubated with the anti-Kir5.1 and anti-GAPDH primary antibodies followed by peroxidase-conjugated goat anti-rabbit and goat anti-mouse antibodies. The SuperSignal West Dura chemiluminescent substrate kit (Thermo Scientific, 34580) was used to visualize the target proteins. Semi-quantification of the Western blot band intensities was performed using Image J software, and the level of each protein was normalized to the GAPDH intensity.

### Cell and Synapse Number Analysis

To quantify the immunopositive cells, the whole-mount cochlear sensory epithelia were divided into three turns. Under the same z-stack conditions with 1-μm intervals, samples were magnified 63× to obtain the image and immunolabeled CtBP2 and PSD95 puncta in IHCs were counted. Based on the presence of the overlapping CtBP2 and PSD95 signals, the number of synapses was counted from the apical turn to the basal turn of the sensory epithelia.

### Statistical Analysis

All experiments were repeated at least three times, and all data are presented as the mean ± SD. Microsoft Excel and GraphPad Prism 8 software were used for statistical analyses. The number “n” in each assay corresponds to the number of independent animals analyzed in each group. Two-tailed and unpaired Student’s *t*-tests were used to determine the statistical differences between two groups, while one-way ANOVA followed by a Dunnett’s multiple comparisons test was used to compare more than two groups. A value of *p* < 0.05 was considered statistically significant.

## Data Availability Statement

The raw data supporting the conclusions of this article will be made available by the authors, without undue reservation.

## Ethics Statement

The animal study was reviewed and approved by Institutional Animal Care and Use Committee of Southeast University. Written informed consent was obtained from the owners for the participation of their animals in this study.

## Author Contributions

JLv and XF contributed equally to this work. BG, HL, YH, and RC conceived and designed the experiments. JLv, XF, YL, GH, JLi, RY, and YX performed the experiments. JLv, XF, LF, WW, and G-LL analyzed the data. JLv and XF wrote the manuscript. All authors contributed to the article and approved the submitted version.

## Conflict of Interest

The authors declare that the research was conducted in the absence of any commercial or financial relationships that could be construed as a potential conflict of interest.
