# Supplementary figures and images for: Deletion of Kcnj16 in Mice Does Not Alter Auditory Function
Source: Front Cell Dev Biol. 2021 Feb 22;9:630361. doi: 10.3389/fcell.2021.630361 (PMC7937937; doi:10.3389/fcell.2021.630361)

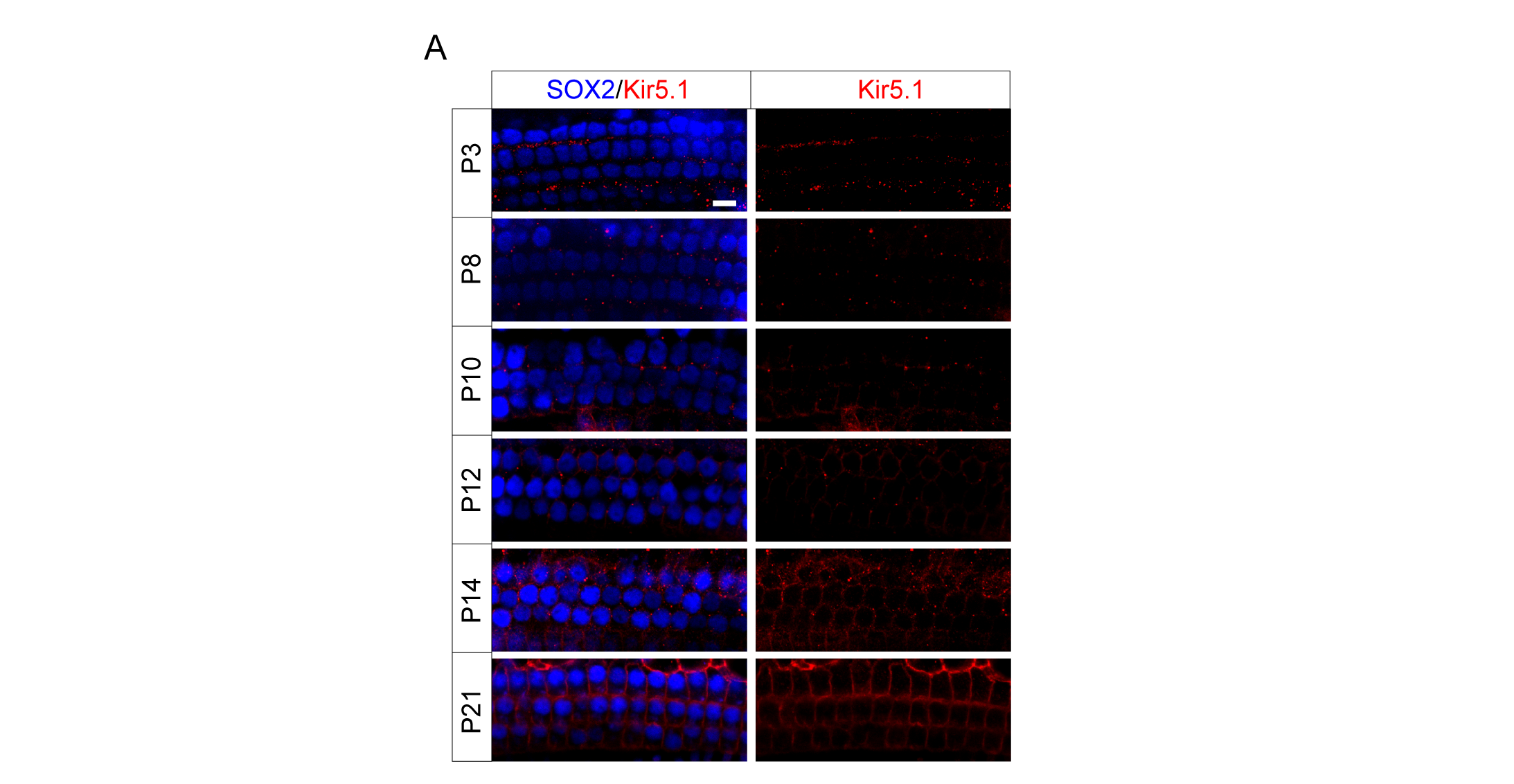

Supplement: Supplementary file 1 [file Image_1.TIF]

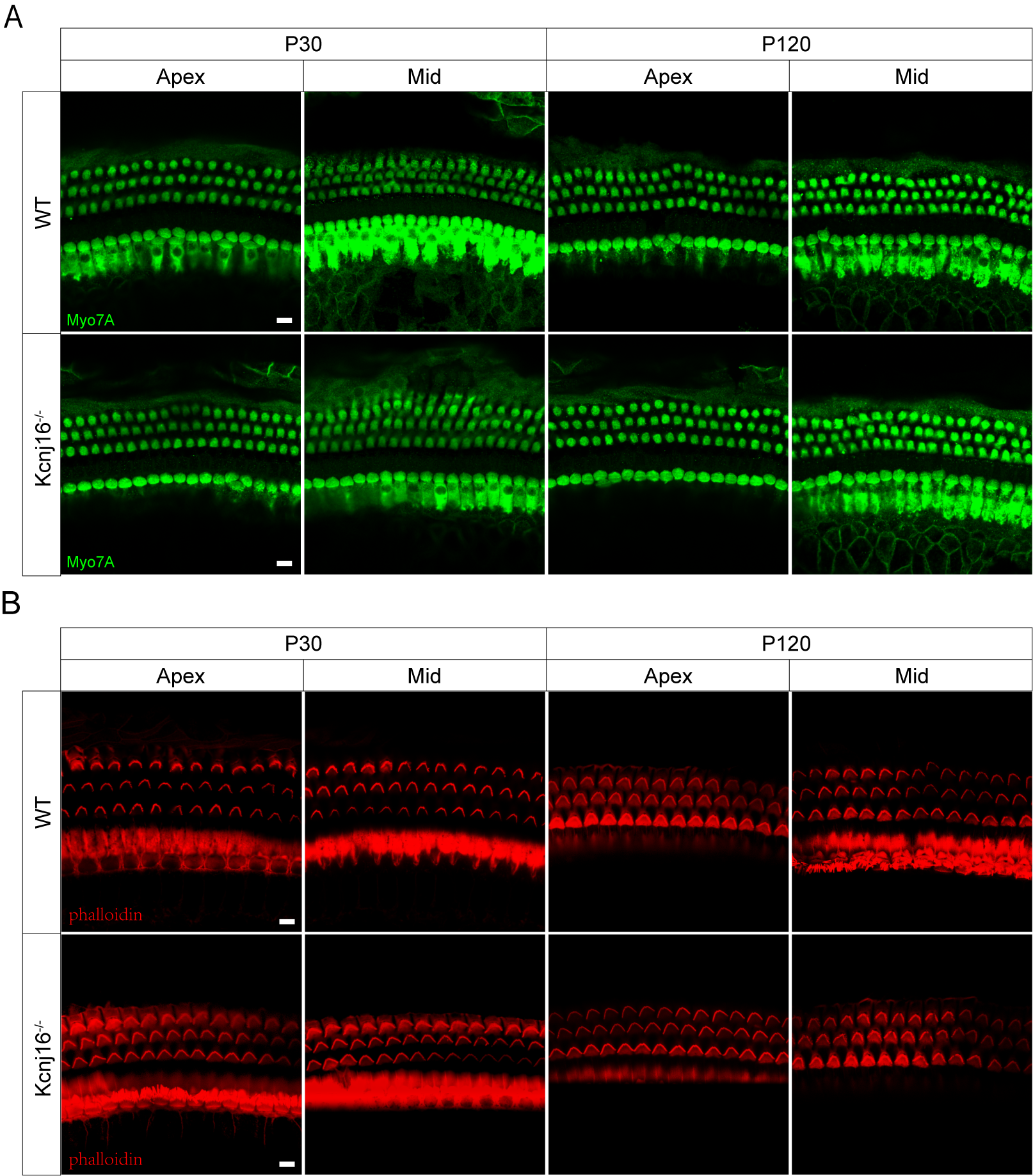

Supplement: Supplementary file 2 [file Image_2.TIF]

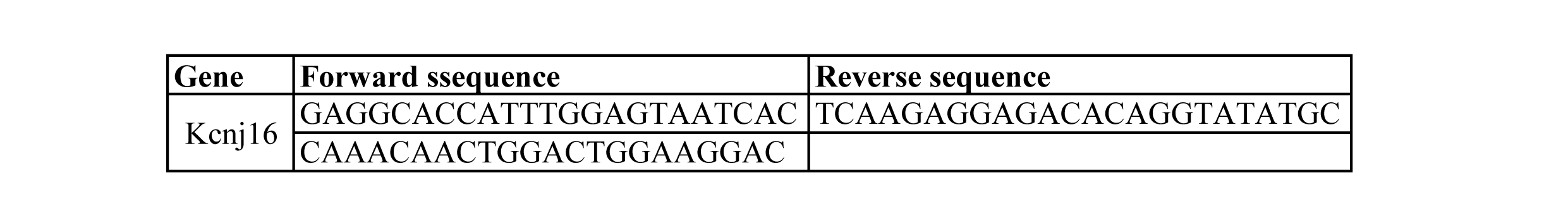

Supplement: Supplementary file 3 [file Image_3.TIF]

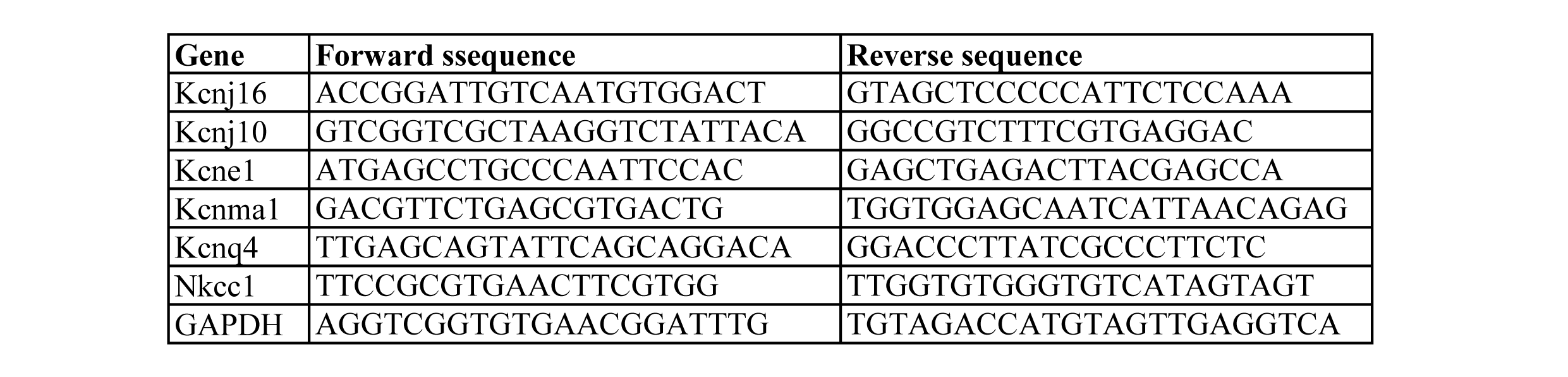

Supplement: Supplementary file 4 [file Image_4.TIF]
